# Racial Disparities in Utilization of Medications and Disease Outcomes in Inflammatory Bowel Disease Patients

**DOI:** 10.1093/crocol/otaf021

**Published:** 2025-03-16

**Authors:** Khaled Alsabbagh Alchirazi, Osama Hamid, Thabet Qapaja, Mohammad Aldiabat, Nour Azzouz, Motasem Alkhayyat, Miguel Regueiro

**Affiliations:** Department of Gastroenterology, Aurora Health Care, Milwaukee, WI, USA; Department of Gastroenterology and Hepatology, University of Texas Southwestern Medical Center, Dallas, TX, USA; Department of Hospital Medicine, Washington University in St. Louis, St. Louis, MO, USA; Department of Hospital Medicine, Washington University in St. Louis, St. Louis, MO, USA; Department of Gastroenterology and Hepatology, Cleveland Clinic, Cleveland, OH, USA; Department of Gastroenterology and Hepatology, Indiana University, Indianapolis, IN, USA; Department of Gastroenterology and Hepatology, Cleveland Clinic, Cleveland, OH, USA

**Keywords:** inflammatory bowel disease, African American, Black, Crohn’s disease, race, ulcerative colitis, racial disparity

## Abstract

**Background:**

Although traditionally associated with White European ancestry, inflammatory bowel disease (IBD) has increased among different races and ethnicities. Large studies conducted in the United States and Canada have identified more complex disease phenotypes among Black patients. Our study aimed to investigate disparities in IBD treatments and outcomes between Black and White patients in the United States.

**Methods:**

Using the TriNetX database, adult IBD patients were divided into 2 groups based on race: Black and White patients with IBD, Crohn’s disease (CD), or ulcerative colitis (UC). Medical therapy and disease outcomes were evaluated in both groups with 1:1 propensity-score matching. Methodologic limitations include the potential for missing data, lack of information on socioeconomic strata, and patient-level medication coverage plans.

**Results:**

In comparison to White patients, Black patients with CD were less likely to receive advanced therapies; Adalimumab (adjusted odds ratio- aOR 0.89), Certolizumab (0.81), Vedolizumab (0.66), Ustekinumab (0.82), or Tofacitinib (0.58). Black patients with UC were less likely to receive advanced therapies; Adalimumab (0.83), Golimumab (0.62), Vedolizumab (0.69), Ustekinumab (0.73), or Tofacitinib (0.55). Black patients with IBD were at higher odds of utilizing corticosteroids (CD 1.18 and UC 1.20) and opioids (CD 1.26 and UC 1.09). Black patients with CD had higher rates of hospitalization (1.35) and perianal abscess (1.56), perianal fistula (1.28), and intestinal fistula (1.38). Black patients with UC had higher rates of hospitalization (1.29), Clostridioides difficile infection (1.11), and toxic megacolon (1.34).

**Conclusions:**

There were racial disparities in IBD medical therapy and disease outcomes. Black IBD patients had lower treatment with advanced therapies, higher opioid and corticosteroid use, and higher IBD-related complications.

## Graphical Abstract

**Graphical Abstract:**
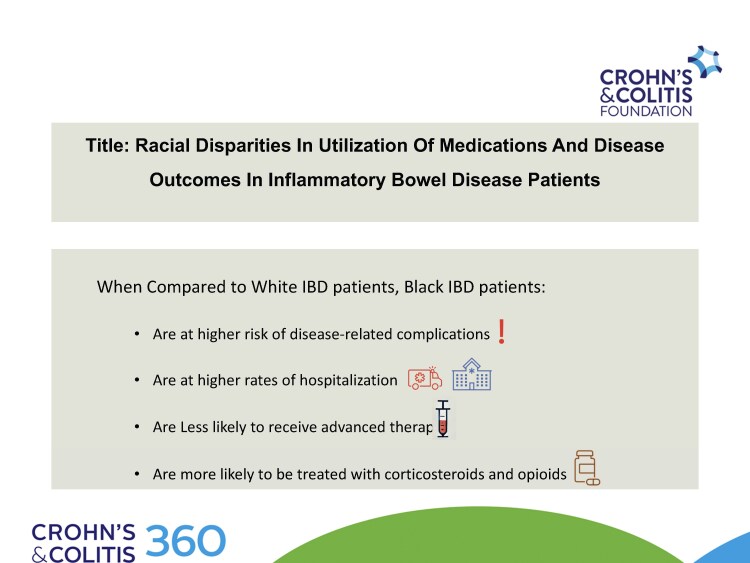

## Introduction

Inflammatory bowel disease (IBD), including Crohn’s disease (CD) and ulcerative colitis (UC), is a chronic inflammatory disorder of the gastrointestinal tract that affects millions of people worldwide.^1^ Although IBD was previously believed to predominantly affect White patients in Europe and North America, recent studies have shown a worldwide rise in CD and UC rates among populations with previously low incidence.^2^ Additionally, emerging literature suggests that genetic determinants of IBD may vary by race, which could lead to phenotypic differences in disease expression.^3^ For example, Black patients may have higher rates of perianal disease,^4^ penetrating disease,^5^ upper gastrointestinal tract CD,^6^ and proctitis or left-sided UC.^7^ Moreover, previous studies have indicated that Black patients may experience more frequent IBD complications, such as hospitalizations and mortality, compared to White and Hispanic populations.^8^ One study found that Black IBD patients had lower access to specialists compared to White patients, and greater concerns related to cost of care.^9^ Recent research has shown increasing disease prevalence and incidence among minority populations, including black patients, highlighting the importance of studying racial disparities in IBD.^10^ The contribution of genetics to the phenotypic variation observed in black IBD patients has also been a focus of recent studies.^11^

It is not known whether there are racial disparities in utilization of advanced IBD therapies. Our study aimed to evaluate differences in IBD treatments and disease outcomes between Black and White patients in a large United States database.

## Materials and Methods

### Database

We performed a cross-sectional analysis of IBD patients using TriNetX, a national healthcare research network, including over 106 million patients, sourced from 73 healthcare organizations (HCO) located within the United States. The participating HCOs include large academic or research-oriented health centers with inpatient, outpatient, and specialty care services.^12,13^ TriNetX (Cambridge, MA, USA) is a global federated health research network that is HIPPA compliant, exempt from IRB, and continuously aggregates clinical data directly from the electronic medical records of participating HCOs. There is extensive data quality and accuracy assessment. TriNetX does not provide institutional details on participating HCOs, provides only de-identified data, and is exempt from approval by the Cleveland Clinic Institutional Review Board.

### Patient Selection

A real-time search and analysis of the US Collaborative Network in the TriNetX platform was conducted and updated through February 18th, 2023. TriNetX analyzes patient data up to 20 years prior to the date of analysis. We identified all adult patients (aged ≥ 18 years) who had at least 2 International Classification of Disease, Tenth Revision, Clinical Modification (ICD-10-CM) codes in their EHR for UC (K51*) or CD (K50*). We also identified IBD medications using Rxnorm codes for any one of the following medications: mesalamine, infliximab, adalimumab, certolizumab, golimumab, vedolizumab, ustekinumab, tofacitinib, azathioprine, mercaptopurine, methotrexate, prednisone, budesonide or opioids. At the time of this analysis, risankizumab and upadicitinib were not commercially available in the United States. Supplementary Table 1. Finally, we stratified this patient population into 2 cohorts, based on race (White and Black patients). We collected cross-sectional information on patient demographics such as gender, age, and comorbidities. Table 1.

**Table 1.** Demographic characteristics of inflammatory bowel disease patients before and after propensity score matching.

Demographics	Before propensity-score matching			After propensity-score matching		
	Black with CD	White with CD	*P* value	Black with CD	White with CD	*P* value
Age at index	41.6 +/− 17.4	45.7 +/− 19	<.001	41.6 +/− 17.4	41.5 +/− 17.5	.825
Female	14 387 (59.0%)	116 933 (56.6%)	<.001	14 386 (59.0%)	14 479 (59.4%)	.392
Male	9995 (41.0%)	89 768 (43.4%)	<.001	9995 (41.0%)	9905 (40.6%)	.407
Essential hypertension	5280 (21.7%)	33 009 (16.0%)	<.001	5279 (21.6%)	5269 (21.6%)	.912
Diabetes mellitus	2835 (11.6%)	16 547 (8.0%)	<.001	2834 (11.6%)	2862 (11.7%)	.693
Hyperlipidemia	2224 (9.1%)	19 628 (9.5%)	.058	2224 (9.1%)	2199 (9.0%)	.693
Chronic kidney disease	1461 (6.0%)	8754 (4.2%)	<.001	1460 (6.0%)	1378 (5.7%)	.113
Heart failure	1077 (4.4%)	6256 (3.0%)	<.001	1076 (4.4%)	952 (3.9%)	.005
Alcohol abuse	569 (2.3%)	3526 (1.7%)	<.001	569 (2.3%)	530 (2.2%)	.234
Nicotine dependence	2269 (9.3%)	14 507 (7.0%)	<.001	2269 (9.3%)	2280 (9.3%)	.864
Cirrhosis	277 (1.1%)	2416 (1.2%)	.651	277 (1.1%)	238 (1.0%)	.084
Demographics	Before propensity-score matching			After propensity-score matching		
	Black with UC	White with UC	*P* value	Black with UC	White with UC	*P* value
Age at index	47.7 +/− 17.8	51.1 +/− 19.1	<.001	47.7 +/− 17.8	47.7 +/− 17.9	.001
Female	11 718 (58.6%)	107 027 (54.2%)	<.001	11 718 (58.6%)	11 767 (58.8%)	.619
Male	8282 (41.4%)	90 343 (45.8%)	<.001	8282 (41.4%)	8235 (41.2%)	.633
Essential hypertension	6723 (33.6%)	44 233 (22.4%)	<.001	6723 (33.6%)	6752 (33.8%)	.759
Diabetes mellitus	3546 (17.7%)	20 201 (10.2%)	<.001	3546 (17.7%)	3566 (17.8%)	.794
Hyperlipidemia	3470 (17.3%)	29 253 (14.8%)	<.001	3470 (17.3%)	3445 (17.2%)	.741
Chronic kidney disease	2063 (10.3%)	10 528 (5.3%)	<.001	2063 (10.3%)	1984 (9.9%)	.190
Heart failure	1379 (6.9%)	8238 (4.2%)	<.001	1379 (6.9%)	1276 (6.4%)	.039
Alcohol abuse	624 (3.1%)	3539 (1.8%)	<.001	624 (3.1%)	583 (2.9%)	.231
Nicotine dependence	2547 (12.7%)	15 085 (7.6%)	<.001	2547 (12.7%)	2595 (13.0%)	.473
Cirrhosis	432 (2.2%)	3037 (1.5%)	<.001	432 (2.2%)	389 (1.9%)	.129

### Study Outcomes

The primary outcome was the racial disparity in IBD medication utilization between Black and White patients with either CD or UC. The secondary outcome was the occurrence of IBD-related complications in Black patients with either CD or UC compared to White patients with either CD or UC. IBD-related complications included hospitalization, perianal abscess, perianal fistula, intestinal obstruction, intestinal fistula, Clostridioides difficile infection, colon cancer, colectomy, and toxic megacolon.

### Statistical Analysis

We collected clinical data including patient demographics, comorbidities, and IBD-related medications. To address potential confounders that could bias our results, we balanced cohorts using 1:1 propensity score matching based on age and gender categories. (Table 1). For continuous data, we performed independent *t*-tests. For categorical data (presented as frequencies and percentages), we performed chi-square tests. For outcomes, we used odds ratios to compare the rates between cohorts. To safeguard protected health information (PHI), TriNetX rounds up patient counts that are less than 10, up to 10. This rounding may affect our measures of association for variables with small patient counts. All tests were two-tailed with an alpha level of 0.05 for statistical significance.

## Results:

### Characteristics of Study Population

Out of the approximately 25 million patients in the database, there were a total of 321 080 patients with CD and 311 235 patients with UC. Before propensity score matching, there were 24 904 Black patients with CD and 212 311 White patients with CD. White patients with CD were older (45.7 +/− 19.1) compared with Black patients with CD (41.6 +/− 17.4), *P* value < .001. Black patients with CD were more likely to be female (59% vs 56.6 %, *P* value < .001). Black and White cohorts with CD were balanced after propensity score matching (*n* = 24 386). In the UC group, there were 20 422 Black patients with UC and 202 269 White patients with UC before propensity score matching. White patients with UC were older (51.1 +/− 19.1) compared to Black patients with UC (47.7 +/− 18.8), *P* value < .001. When compared with White patients with UC, Black patients with UC were more likely to be female (58.6 % vs 54.2%, *P* < .001). Black and White cohorts with UC were balanced after propensity score matching (*n* = 20 004). The baseline demographics and comorbidities of the study population were stratified by race and reported before and after propensity matching (Table 1).

### Medication Utilization Between Black and White Patients With IBD

**Figure 1.**
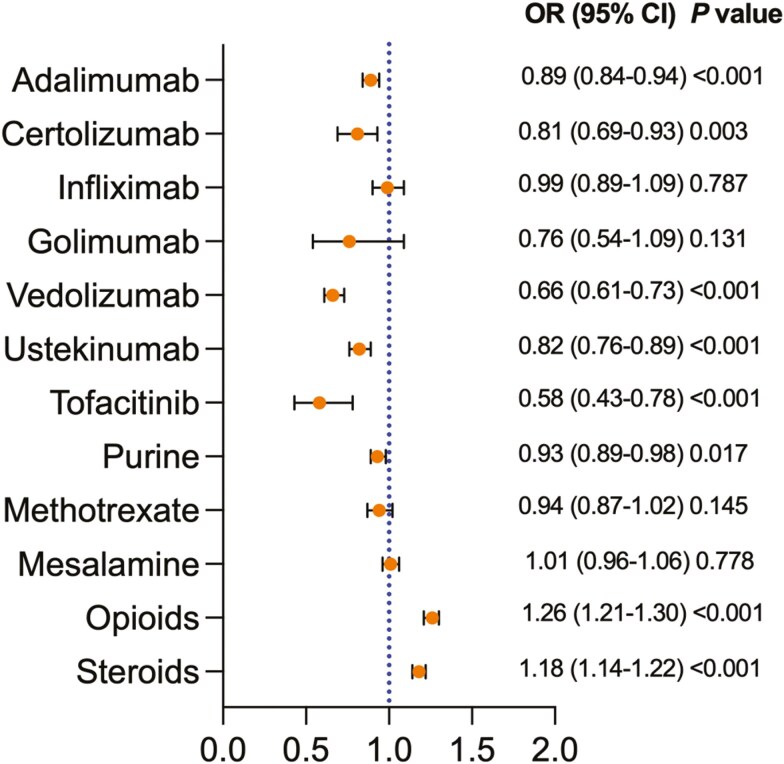
Medication Use between Black Patients With CD Compared With White Patients With CD after propensity score matching. OR, odds ratio; CI, confidence interval, Purine; azathioprine and mercaptopurine.

Black CD patients were less likely than White patients to be treated with adalimumab (OR 0.89, 95% CI: 0.84-0.94) and certolizumab (OR 0.81, 95% CI: 0.69-0.93). There were no significant differences between the 2 cohorts in utilizing infliximab (OR 0.99, 95% CI: 0.89-1.09) or golimumab (OR 0.76, 95% CI: 0.54-1.09). Black patients with CD were less likely to be treated with other advanced therapies and thiopurines: vedolizumab (OR 0.66, 95% CI: 0.61-0.73) ustekinumab (OR 0.82, 95% CI: 0.76-0.89), tofacitinib (OR 0.58, 95% CI: 0.43-0.78), and thiopurines (OR 0.93, 95% CI: 0.89-0.98) compared with White patients. There were no significant differences between the 2 cohorts in methotrexate (OR 0.94, 95% CI 0.87-1.02) and mesalamine (OR 1.01, 95% CI: 0.96-1.06) utilization. Opioids and corticosteroid utilization were significantly higher in Black patients compared with White patients (OR 1.26, 95% CI: 1.21-1.30, and OR 1.18, 95% CI: 1.14-1.22, respectively), Figure 1.

**Figure 2.**
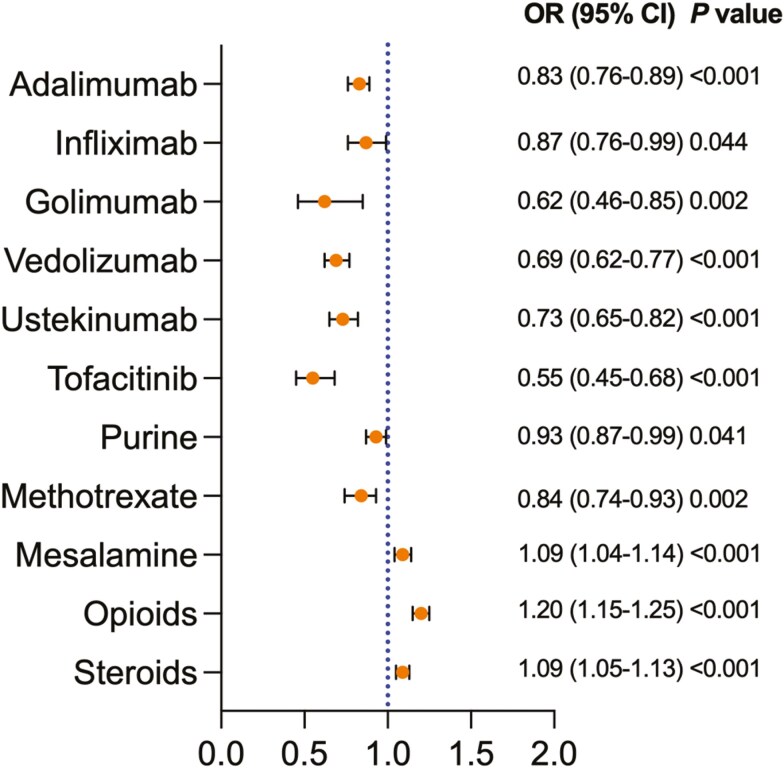
Medication Use between Black Patients With UC Compared With White Patients With UC after propensity score matching. OR, odds ratio; CI, confidence interval, Purine; azathioprine and mercaptopurine.

Black patients with UC were less likely than White patients to be treated with any of the advanced therapies: infliximab (OR 0.87, 95% CI: 0.76-0.99), adalimumab (OR 0.83, 95% CI: 0.76-0.89), golimumab (OR 0.62, 95% CI: 0.46-0.85), vedolizumab (OR 0.69, 95% CI: 0.62-0.77), ustekinumab (OR 0.73, 95% CI: 0.65-0.82) and tofacitinib (OR 0.55, 95% CI: 0.45-0.68). Opioids and corticosteroids treatment was higher in Black patients compared with White patients with UC (OR 1.20, 95% CI: 1.15-1.25 and OR 1.18, 95% CI: 1.14-1.22, respectively), Figure 2.

### IBD-Related Outcomes in Black and White Patients

**Figure 3:**
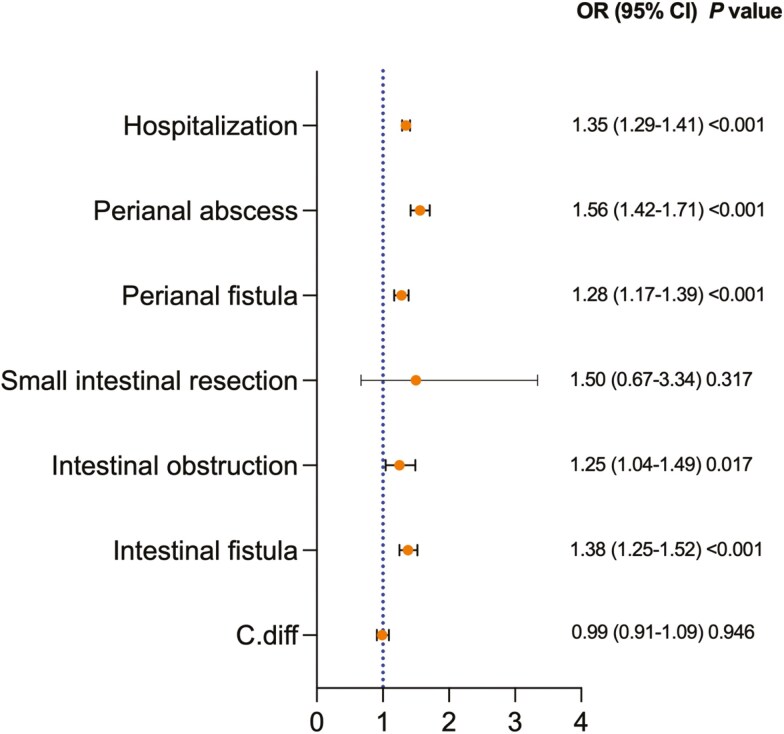
Crohn’s disease (CD) related complication between Black Patients With CD Compared With White Patients With CD after propensity score matching. OR; odds ratio. CI; confidence interval. OR; odds ratio. CI; confidence interval. C.diff; Clostridioides difficile.

Among patients with CD, Black patients had higher rates of hospitalization (OR 1.35 95% CI: 1.29-1.41), intestinal obstruction (OR 1.25, 95% CI: 1.04-1.49), intestinal fistula (OR 1.38, 95% CI: 1.25-1.52), perianal abscess (OR 1.56, 95% CI: 1.42-1.71) and perianal fistula (OR 1.28, 95% CI: 1.17-1.39) compared to White patients. There were no significant differences in small intestinal resection (OR 1.5, 95% CI: 0.67-3.34) or Clostridioides difficile infection (OR 0.99, 95% CI: 0.91-1.09) when we compared both cohorts. Figure 3.

**Figure 4:**
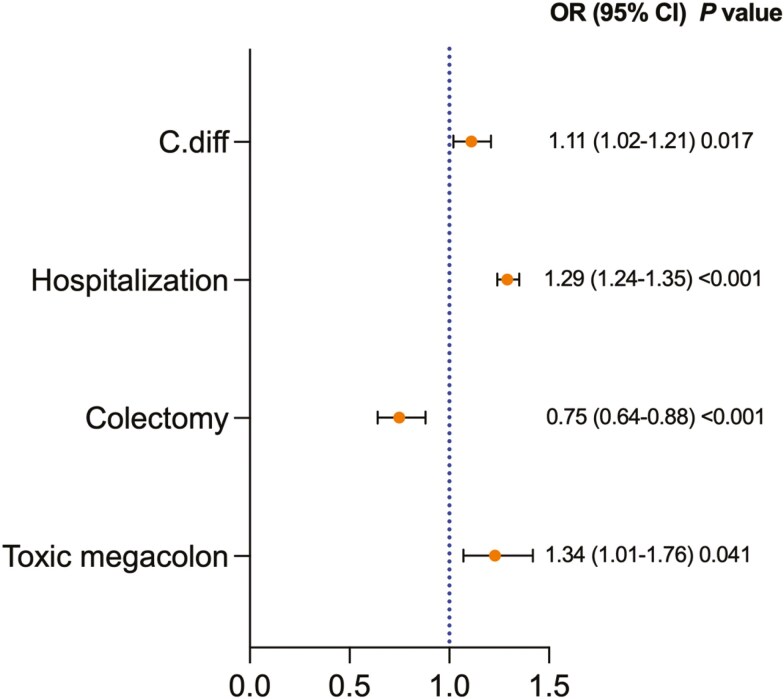
Ulcerative colitis (UC) related complication between Black Patients With UC Compared With White Patients With UC after propensity score matching. OR; odds ratio. CI; confidence interval. C.diff; Clostridioides difficile.

Compared to White patients, Black patients with UC had higher rates of hospitalization (OR 1.29, 95% CI: 1.24-1.35), toxic megacolon (OR 1.34, 95% CI: 1.01-1.76), C.diff (OR 1.11, 95% CI: 1.02-1.21), but lower rates of colectomy (OR 0.75, 95% CI: 0.64-0.88). Figure 4.

## Discussion

Our study showed that Black IBD patients were less likely to receive advanced therapies, but more likely to receive corticosteroids and opioids compared to White patients. Black patients were also more likely to be hospitalized and develop IBD-related complications.

Although Black and White patients with CD did not differ in their use of infliximab, UC Black patients were less likely to receive infliximab. Infliximab for CD was the only therapy that had similar utilization rates between Black and White patients. This finding was similar to a cohort study by Preesman et al found no racial disparity in the utilization of infliximab between Black and White patients.^14^ Similarly, Barnes et al showed no difference in utilization of anti-TNF therapies between races.^4^ The nature of our database could not assess why there was a similarity in infliximab usage between Blacks and Whites in CD. Infliximab is the biologic that has been on the market the longest for CD, and it is possible that this provided similar access for all patients. Our finding that Black patients had lower rates of utilization of infliximab in UC is consistent with prior publications. Nguyen et al found that Black patients were less likely to receive infliximab (41% vs. 60%, *P* = .01).^9^ Our findings were also similar to those of Flasar et al who reported a lower frequency immunomodulator, and infliximab use in Blacks (RR 0.51, 95% CI: 0.27-0.96).^15^ Recent systematic reviews by Sewell et al and Afzali et al, both showed decreased use of anti-TNFs in Black patients.^16,17^

Our study also evaluated newer biological therapies and included the first small molecule approved for IBD. For all of these medications, we found that Black patients were less likely to receive integrin receptor antagonist (Vedolizumab), anti-IL 12/23 antibody (Ustekinumab), or janus kinase inhibitor (Tofacitinib).

Despite a lower utilization of advanced therapies, we found increased utilization of steroids and opioids in Black patients. Both steroids and opioids have been associated with increased IBD complications, morbidity, and mortality.^18,19^ Guidelines and consensus statements recommend against the long-term or repeated use of corticosteroids and for the control of pain with non-opioid analgesics.^20^ The reason for a decreased utilization of advanced therapies and increased utilization of corticosteroids and opioids in our study is not clear but could be due to lack of access to specialized IBD care and cost of medications, ie, under-insured or uninsured. A national inpatient sample study from 2007 concluded that Black IBD patients were more likely to have lower incomes than the national average. In addition, Black patients were more likely to have Medicaid, be underinsured, or uninsured in comparison to the White population.^21–23^

According to Nguyen et al, Black patients were less likely than Whites to receive regular care from gastroenterologists or IBD specialists.^9^ Barriers to access IBD care were explored by Straus et al who reported that Black patients had difficulty affording health care, postponed appointments to specialist care due to financial concerns, and found travel to providers’ offices prohibitive.^24,25^ One study found higher IBD-related emergency department visits in Black patients.^9^ Frequent unplanned care, ie, ED visits and hospitalizations, has been linked to higher use of opioid medications in IBD.^26^ Our study did find higher rates of unplanned care, specifically hospitalizations, and would represent a possible explanation for higher rates of opioid use in Black patients.

CD-related complications, such as perianal fistula, abscesses, intestinal fistula, and small bowel obstruction were higher in Black patients compared to White patients. Our findings are similar to multiple prior studies of higher prevalence of perianal disease in the Black population than in White (31% vs 14%, *P* = .02), and higher rates of fistulizing perianal CD in Blacks than in White (OR 2.63, *P* < .001).^6,27,28^ A 10-year retrospective analysis by Eidelwein et al found that Black pediatric patients with IBD were more likely to have stricturing and penetrating CD, lower hemoglobin levels, and receive more corticosteroids and infliximab in their disease course, signifying more severe disease course when compared to Whites.^29^ There have been other studies which describe variations in environmental exposure or genetic polymorphisms as a possible reason for phenotypic differences between Black and White IBD populations^2,3,30^ Whether the Crohn’s related complications that we found in our study were related to undertreatment with advanced therapies is not clear. The study by Sewell et al did suggest an impact of race and socioeconomic disparities on treatment modalities and healthcare delivery.^16^

Our UC cohort showed a higher rate of Clostridioides difficile infection in Black patients compared to Whites. Racial differences in Clostridioides difficile infection have been previously described. Although not specifically studied in IBD, the Clostridioides difficile infection differences may be related to disparity in healthcare access and utilization of antibiotics.^31,32^ Black patients with Clostridioides difficile infection also had higher mortality rates and more severe infection.^33–35^ The causality for the higher rates of Clostridioides difficile infection in Black patients in our study could not be determined. In prior studies, Clostridioides difficile infection has been linked to uncontrolled inflammation and more severe UC.^36,37^ To that end, it is plausible that uncontrolled UC is an explanation.

Our study has limitations. TrinetX is a database that provides a selection of electronic health record (EHR) systems-derived data. The primary purpose for collecting the data in EHR systems is to offer clinical care, assign billing codes, and meet relevant regulatory obligations. Consequently, the data may be subject to selection bias, coding input errors, missing data, and follow-up biases. One limitation of our study is the use of a case definition based on ≥1 ICD-10-CM code without requiring IBD-related prescriptions. This approach was chosen to avoid incorporating medication use as part of the identification criteria, as it was a primary outcome in the comparison between racial groups. Furthermore, the observed disparity in the number of Black and White patients with IBD may reflect underdiagnosis, disparities in healthcare access, or biases in reporting and coding practices. A further limitation is that the database lacks detailed ethnicity data, such as distinguishing between non-Hispanic and Hispanic White individuals, which constrains the interpretation of racial disparities. Additionally, while TrinetX captures prescriptions, it does not provide information on patient compliance with treatments or treatment coverage, such as out-of-pocket payments, government-subsidized programs, or specific insurance plans.

However, TrinetX utilizes data from source systems and subjects it to transformation, cleanup, deduplication, deidentification, optional obfuscation, and semantic mapping before use, thereby limiting potential biases and data errors.^38^ The retrospective design of our study is another limitation, as it lacks specific information about disease course, IBD severity, disease location and behavior, and history of extra-intestinal manifestations. Moreover, the correlation of certain outcomes may not always be attributable to IBD. Importantly, the database does not include crucial contextual factors such as patient preferences, cultural beliefs, socioeconomic status, education level, or insurance status. Presumably, some of these factors could explain some, but not all, healthcare disparities.

In conclusion, we found racial disparities in medication utilization and disease outcomes among Black and White IBD patients in the United States. There was lower prescribing of advanced therapies, but higher of corticosteroids and opioids in Black patients. IBD-related complications were also higher in Black patients. Our study further elucidates the need to identify and address the healthcare barriers that disproportionately affect Black patients with IBD.

## Supplementary Material

otaf021_suppl_Supplementary_Tables_S1

## Data Availability

Data are available upon reasonable request from the corresponding author.
