# Supplementary material for: Racial Disparities in Utilization of Medications and Disease Outcomes in Inflammatory Bowel Disease Patients
Source: Crohns Colitis 360. 2025 Mar 16;7(2):otaf021. doi: 10.1093/crocol/otaf021 (PMC12010087; doi:10.1093/crocol/otaf021)
Supplement: otaf021_suppl_Supplementary_Tables_S1 [file otaf021_suppl_supplementary_tables_s1.docx]

**Supplementary table 1:** A complete list of ICD-10-CM, CPT, and RxNorm codes

| **Cohort** | **Terms** | **ICD-10/CPT/RxNorm Codes** |
| --- | --- | --- |
| IBD | Ulcerative colitis | K51 |
|  | Crohn’s disease | K50 |
|  |  |  |
| IBD medication | Prednisone | 8640 |
|  | Budesonide | 19831 |
|  | Mesalamine | 52582 |
|  | Azathioprine | 1256 |
|  | Mercaptopurine | 103 |
|  | Methotrexate | 6851 |
|  | Infliximab | 191831 |
|  | Adalimumab | 327361 |
|  | Certolizumab | 709271 |
|  | Golimumab | 819300 |
|  | Vedolizumab | 1538097 |
|  | Ustekinumab | 847083 |
|  | Tofacitinib | 1357536 |
|  |  |  |
| IBD related complication | Perianal fistula | K60xx |
|  | Perianal abscess | K61xx |
|  | Clostridioides difficile | A047xx |
|  | Intestinal obstruction | K56.5 |
|  | Intestinal fistula | K63.2 |
|  | Intestinal resection | Z90.49 |
|  | ICU requirement | requiring mechanical ventilation: 5A1935Z, 5A1945Z, 5A1955Z OR extracorporeal membrane oxygenation Z92.81 |
|  | Hospitalization |  |
|  | Mortality |  |
|  | Total abdominal colectomy with end ileostomy: | 0DTE4ZZ, 0DBE4ZZ, 0DBE0ZZ, 0DTE0ZZ |
|  | Status post colectomy | Z90.49 |
|  | Toxic megacolon |  |
|  | Small Intestine resection | 0DT80ZZ |
|  |  |  |
| Comorbidities | Chronic Hypertension | I10x, I15x, I16x, O100x, O104x,  O109x, O11x, O16x |
|  | Smoking | F172xx, Z720, Z87891 |
|  | Alcohol abuse | F10XXX |
|  | Diabetes mellitus | E08-E13 |
|  | Hyperlipidemia | E78.5 |
|  | Congestive heart failure | I110, I130, I50XXX |
|  | Chronic kidney disease | N18X, N19 |
|  | Congestive Heart Failure | I110, I130, I50XXX |

IBD: Inflammatory bowel disease.
